# Vaso-occlusive crisis in sickle cell disease: a vicious cycle of secondary events

**DOI:** 10.1186/s12967-021-03074-z

**Published:** 2021-09-20

**Authors:** Tim Jang, Maria Poplawska, Emanuela Cimpeanu, George Mo, Dibyendu Dutta, Seah H. Lim

**Affiliations:** Division of Hematology and Oncology, Department of Medicine, SUNY Downstate Medical Center, SUNY Downstate Health Sciences University, 450 Clarkson Avenue, MSC #20, Brooklyn, NY 11203 USA

**Keywords:** Sickle cell disease, Secondary vaso-occlusive crisis, Downstream events, Treatment strategies

## Abstract

Painful vaso-occlusive crisis (VOC) remains the most common reason for presenting to the Emergency Department and hospitalization in patients with sickle cell disease (SCD). Although two new agents have been approved by the Food and Drug Administration for treating SCD, they both target to reduce the frequency of VOC. Results from studies investigating various approaches to treat and shorten VOC have so far been generally disappointing. In this paper, we will summarize the complex pathophysiology and downstream events of VOC and discuss the likely reasons for the disappointing results using monotherapy. We will put forward the rationale for exploring some of the currently available agents to either protect erythrocytes un-involved in the hemoglobin polymerization process from sickling induced by the secondary events, or a multipronged combination approach that targets the complex downstream pathways of VOC.

## Background

Sickle cell disease (SCD) is a genetic disorder of the hemoglobin (Hb) affecting ~30 million people worldwide [1]. It is characterized by a point mutation that results in the substitution of glutamic acid with valine on the sixth amino acid of β-globin. The mutant Hb molecules, hemoglobin S (HbS), polymerize upon deoxygenations, causing the erythrocytes to assume a sickle morphology and reduce the fluidity of the cell membrane. Sickle erythrocytes adhere to leukocytes immobilized to the endothelium, causing microvascular occlusion, vaso-occlusive crisis (VOC), and tissue ischemia. Recurrent vaso-occlusion causes chronic disabling arthritis due to osteonecrosis affecting the joints, progressive retinopathy, chronic renal failure, increased risks for strokes, and shortened lifespan.

In the United States, although only 100,000 Americans are affected by the disease [1], they impose high healthcare utilization [2]. In 2004, more than 80,000 hospitalizations were incurred by adult SCD patients in the US, costing nearly $500 million, with majority of the cost arising from inpatient hospitalization associated with VOC [3]. A benchmark study found a mean annual rate of 1.52 admissions per SCD patients [3]. Efforts in the Emergency Department (ED) to reduce the number of hospitalizations has met with limited successes. A study involving nearly 40,000 ED visits for VOC found that more than 40% of the treat-and-release visits had either an inpatient hospitalization or another ED treat-and-release visit within 14 days of the index ED visit [4].

VOC has previously been suggested to consist of four phases [5, 6]. Phase 1 spans ~3 days and is associated with a low-intensity aching pain. The patient may also report numbness and paresthesia. The aching increases rapidly in Phase 2, to maximum pain due to local tissue infarct from vaso-occlusion. Phase 3 of VOC is due to post-infarct inflammatory responses and is when the severe pain becomes constant and this may be associated with a fever. Phase 3 usually lasts three to five days. This is followed by the resolution of the VOC in Phase 4 over one to two days. Although the phasic process of VOC appears straightforward, various clinical observations suggest that VOC is in fact a fluid process that does not always follow this sequence of events. Phases 2/3 are not always followed by Phase 4. Instead, these phases may feedback into Phase 1 to create a vicious cycle of VOC that we previously proposed [7]. Examples supporting this notion are seen in the prolonged hospital length-of-stay (LOS) beyond the average of five days for an episode of VOC [8] in many patients, the development of worsening symptoms, and the onset of acute chest syndrome during hospital stay. We recently found in a study in our institution of more than 100 episodes of uncomplicated VOC admissions that 3% of these patients subsequently developed acute chest syndrome during hospitalization [9]. In patients treat-and-released in ED for painful crisis, there was also a high frequency of subsequent inpatient hospitalization or another ED visit within 2 weeks [4]. Given the complexity of VOC and its positive feedback mechanism that may further exacerbate the pain crisis and to set up a vicious cycle that we now term secondary VOC (sVOC), adequate and aggressive intervention of VOC at the earliest time is crucial. This will break the vicious cycle and shorten hospital LOS due to pain, although the component of pain due to tissue infarct may not be rescuable. Here, we defined sVOC as VOC that arises from the downstream processes caused by the initial VOC episodes. In this paper, we will summarize the mechanisms and pathophysiology of the chronic inflammatory processes associated with SCD, define and examine the molecular events of VOC and how they might promote sVOC, and propose a multipronged approach for the management of VOC episodes to prevent the development of sVOC.

### Chronic inflammatory processes in sickle cell disease

**Fig. 1:**
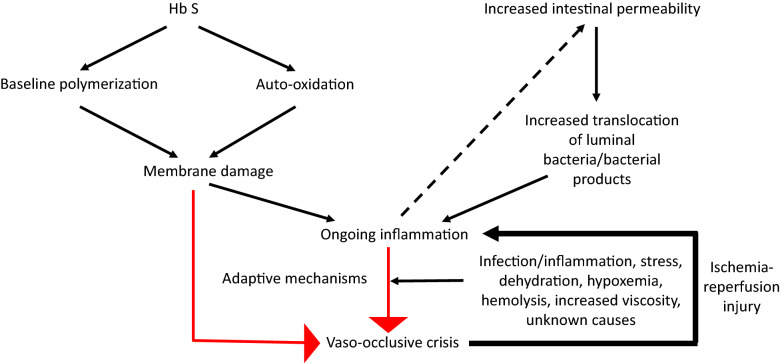
Origins of ongoing inflammatory processes in SCD patients. Endogenous stimuli arise due to erythrocyte membrane damage induced by a combination of background HbS polymerization and auto-oxidation. Exogenous stimuli contributing to the ongoing inflammatory process are due to increased intestinal permeability and enhanced translocation of bacteria/bacterial products into the systemic circulation to stimulate leukocytes and aged neutrophils. The inflammatory processes are compensated by various host adaptive mechanisms that prevent the progress of the process to VOC. Events such as infection/inflammation, stress, dehydration, increased hemolysis, and hypoxemia tilt the equilibrium and result in a decompensation state that precipitates the development of VOC. Cytokine reactions associated with ischemia/reperfusion injury induced by VOC may further feed into increasing the intestinal permeability (represented by dashed lines) and form part of the vicious cycle of vaso-occlusion

In addition to erythrocyte sickling, one of the hallmarks of SCD is the continuous presence of basal inflammatory processes. This is exemplified in the following observations. SCD patients have higher baseline leukocyte counts than those without the disease [10]. Although the elevated leukocytes may be contributed by the overstimulated marrow from the underlying hemolytic process, SCD patients exhibit higher levels of soluble CD62L [11, 12], a marker of neutrophil activation. Neutrophils in SCD also express higher activation molecules, e.g. CD64 [12] and CD11b/CD18 [13]. Monocytes in SCD demonstrate activated phenotypes and higher propensity to IL-1β and TNFα [14]. Similarly, platelets in SCD are chronically activated and express higher level of CD40L [15]. The ongoing inflammatory processes provide the background on which VOC develops. The ongoing inflammatory processes originate from a combination of membrane damage of erythrocytes carrying HbS and increased intestinal permeability that occurs in SCD (Fig. 1). However, once VOC is triggered, ischemia–reperfusion injury that follows further feeds into the inflammatory processes.

### Endogenous stimuli promoting inflammatory processes in sickle cell disease

HbS causes damage to erythrocyte membrane. In addition to background polymerization that affects the fluidity of the membrane, HbS precipitates on the inner surface of the membrane following auto-oxidation and generates iron-dependent free radicals that impact membrane damage [16]. The damaged membrane renders the erythrocytes more adherent to the endothelium although works involving intravital microscopy found that sickle erythrocytes are more prone to adhere to leukocytes immobilized on endothelium than directly to endothelial cells [17].

Before the development of VOC and the consequences of ischemia–reperfusion injury, the hemolysis of erythrocytes in SCD per se promote inflammation. As a result of intravascular hemolysis, free heme is released by the damaged erythrocytes. Free heme activates complements [18]. It mediates pro-inflammatory processes by modulating macrophages via transcription factors such as BTB and CNC homologue (BACH) 1 and Spi-C [19]. Free heme also promotes neutrophil extracellular traps (NETosis) [20]. These ongoing inflammatory processes are, however, kept in equilibrium by various adaptive regulatory mechanisms that include nitric oxide and macrophages. In addition, extracellular heme released from hemolysis bind to plasma heme scavenger proteins such as haptoglobin and undergo further degradation in the liver [21]. These regulatory mechanisms, therefore, keep the processes in a newly established homeostasis and prevent the development of VOC.

### Exogenous stimuli promoting inflammatory processes in sickle cell disease

In addition to the endogenous stimuli, exogenous stimuli in the form of lipopolysaccharides (LPS) also promote the inflammatory processes in SCD. SCD patients exhibit higher level of serum LPS compared to non-SCD patients [11]. LPS not only stimulates and activate neutrophils [22] and monocytes [23], it also regulates aging of neutrophils via its interaction with Myd88 and TLR2/4 [24]. The elevated levels of serum LPS are likely related to increased translocation of bacteria/bacterial products across the intestinal barrier caused by enterocyte injury. The higher levels of serum intestinal fatty acid binding protein (iFABP) detected in the serum of SCD patients would support this notion [11]. Whether the enterocyte injury results in straightforward anatomical defects of the intestinal barrier and/or downregulation of the tight junctions between enterocytes remains to be established. The latter may be supported by the ability of l-glutamine to reduce the frequency of VOC in SCD [25]. It is likely the intestinal microbial load and composition also play a role. SCD patients show intestinal dysbiosis [26]. Antibiotic administration to SCD patients [27] and mice [24] both led to drops in circulating aged neutrophils (CANs), through a combination of reduction in the intestinal microbial load and alteration in the microbial compositions [28]. In SCD patients, there were also associated drops in the serum CD62L [28], indicating a downregulation of the inflammatory processes when the intestinal microbial load and/or composition is altered.

### Ischemia–reperfusion injury

The ongoing inflammatory processes are further worsened if VOC occurs because VOC is invariably followed by ischemia–reperfusion injury. Ischemia–reperfusion injury promotes inflammation by the production of oxygen species that damages local tissues [29]. The damaged tissue increases adhesion and activation of leukocytes, with consequences of cytokine secretion and release of tissue factor that activate the coagulation pathway to promote local thrombotic events [30] that will worsen hypoxemia and provide a positive-feedback to the inflammatory cascade. Ischemia–reperfusion injury also activates invariant Natural killer T (iNKT) cells via CD1d. iNKT cells are hyper-responsive to changes due to reperfusion and secretes chemokines to recruit more leukocytes to the local area of inflammation and worsen tissue damage [31].

### Consequences of chronic inflammatory processes in sickle cell disease

The extent of the balance between chronic inflammatory processes and various adaptive mechanisms that maintain the equilibrium probably varies among different SCD individuals. Susceptibility to developing VOC among different individuals may depend on the size of the adaptive reserves in the compensated state. This will be discussed further in the next section.

Ongoing inflammatory processes not only render SCD patients to developing VOC, they also create a prothrombotic state [32]. In addition, ongoing inflammations create a state of hypermetabolism that may be responsible for the lower appetite and consequent calorie intake [33] and low prevalence of obesity and diabetes mellitus [34] among SCD patients.

### Pathogenesis and molecular events of vaso-occlusive crisis

The mechanism of VOC is complex and yet to be fully dissected. However, it is most likely VOC occurs when the balance between the pathologic processes of SCD responsible for the ongoing inflammation and the hosts adaptive mechanisms is disrupted in favor of the pathologic processes. As a result, the host adaptive mechanisms are overwhelmed, and this leads to decompensation of the balanced state.

**Fig. 2:**
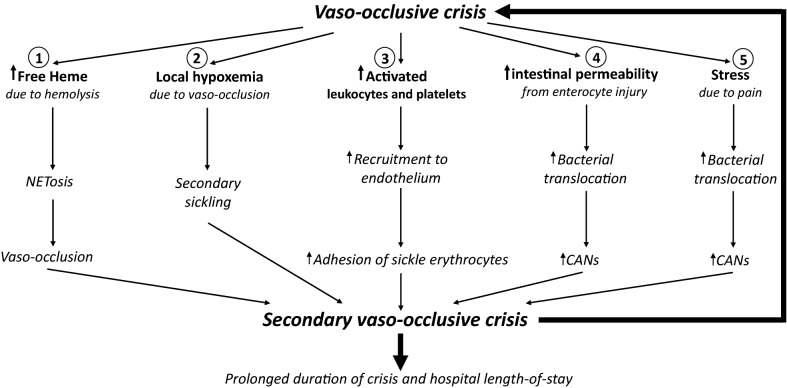
The complex downstream events induced by VOC. Among the many downstream events are the following five pathways, all of which result in a positive-feedback into the VOC process to induce secondary VOC: (1). Increased hemolysis produces more free heme and overwhelms heme scavenging mechanisms, precipitating the development of neutrophil extracellular traps that promotes a local prothrombotic condition. (2). Local hypoxemia caused by vaso-occlusion induces sickling of erythrocytes in the vicinity. (3). Cytokine release due to VOC activates leukocytes and platelets and increases their recruitment to the site of local tissue damage. (4). Increased enterocyte damage due to VOC further increases the gut permeability and worsens the translocation of luminal bacteria/bacterial products into the systemic circulation. (5). Stress induced by painful VOC leads to increased production of epinephrine and glucocorticoid response. The former enhances the expression of adhesion molecules on the erythrocytes and the latter worsens the translocation of luminal bacteria/bacterial products into the systemic circulation

VOC begins with increased sickling and interaction of the sickle erythrocytes with the endothelium in the post-capillary venules, either directly or by way of leukocytes adherent to the endothelium, leading to obstruction of blood flow that triggers a host of signaling cascades. Leukocytes that adhere to the endothelium and form the nidus for sickle erythrocytes to be attached to include activated leukocytes and aged neutrophils. Following the development of vaso-occlusion, several downstream pathologic processes are amplified beyond those that can be held in check by the host adaptive mechanisms. Although the mechanisms are yet to be fully understood, VOC creates a number of distinct downstream processes that are important contributors to the vicious cycle of the complication (Fig. 2).

### Increased release of free heme

When VOC occurs following a precipitating event, the hemolytic process of sickle erythrocytes is increased, with subsequent release of more free heme. Normally, extracellular heme binds to various plasma heme scavenger proteins such as haptoglobin and undergo degradation in the liver [21]. However, due to chronic hemolysis, heme-binding proteins are chronically low in most SCD patients. As a result, free heme accumulates in the system [35]. Elevated levels of free heme are associated with increased incidence of VOC [36]. Free heme stimulates the migration of leukocytes to the endothelial wall where they bind to P- and E-selectins and promote the production of reactive oxygen species (ROS) [37]. Leukocytes immobilized to the endothelium form the nidus for the adherence of sickle erythrocytes, leading to vascular obstruction and local hypoxemia. Vaso-occlusion is also made worse when the activated neutrophils interact with each other to create a network of obstructive strands that consist of neutrophil DNA, histones, proteases, and nucleosomes that are expelled from the decondensed chromatins in the nucleus, a process known as NETosis [38]. Although NETosis is an important host defense system against microorganisms, it incurs injury to the endothelial cells as well as parenchymal tissues across organs [39]. It also stimulates the coagulation cascade [40, 41]. Free heme-mediated pathologic processes create additionally a local microenvironment favoring further sickling of the erythrocytes and the development of sVOC.

### Secondary sickling induced by local hypoxemia and activation of leukocytes and platelets

As a result of VOC, hypoxemia in the vicinity of the vaso-occlusion occurs. Hypoxemia provides the positive feedback to induce polymerization of HbS in erythrocytes that have not previously been involved in the sickling process. Hypoxemia also activates monocytes [42] and platelets [43], and enhances neutrophil inflammatory responses [44]. Both processes facilitate the recruitment and adherence of these activated blood cells to the endothelium to provide the niduses for sickle erythrocytes to be attached to, setting up a vicious cycle of sVOC.

### Increased intestinal permeability

As discussed in the earlier section, SCD patients exhibit increased intestinal permeability [11] that facilitates enhanced translocation of bacteria/bacterial products across the intestinal barrier into the systemic circulation. Increased bacteria/bacterial products in the systemic circulation activates neutrophils and contributes to the ongoing inflammatory processes. In addition, it may also upregulate the expansion of CANs [24]. Unpublished data from our laboratory in experiments using gastric gavage administration of FITC-dextran 4 k to Townes SCD mice found that the intestinal barrier is acutely breached in response to VOC. The increased intestinal permeability is most likely related to enterocyte injury [11], although the pathologic mechanisms responsible for the enterocyte injury remain to be determined. Possible causes include hypoxemic and ischemia–reperfusion injuries from VOC affecting the splanchnic vasculature, direct effects of the inflammatory cytokines on the enterocytes, and intestinal dysbiosis [26]. Both activated and aged neutrophils are highly adherent to endothelial cells and they fuel sVOC.

### Increased stress-induced responses

VOC may induce intense sympathetic responses from stress due to pain. The sympathetic responses result in the release of epinephrine and glucocorticoids. Epinephrine upregulates the expression of intercellular adhesion molecule-4 (ICAM-4) on erythrocytes [45], promoting their binding to endothelial αvβ3 to worsen the VOC. This may explain the pathogenesis of stress being a precipitating factor of VOC [46]. A recent study in mice found that stress-induced VOC is facilitated through intestinal microbiota [47]. Stress leads to augmented glucocorticoid ressponse and subsequent higher gut permeability, which promotes microbiota-dependent interleukin-17A (IL-17A) secretion and increase in circulating pool of CANs. Intestinal microbiota-mediated CAN expansion is mediated particularly by Gram-positive organisms. Stress, therefore, could potentially feed into the vicious cycle of VOC by promoting the development of sVOC.

### Therapeutic agents that may modify acute vaso-occlusive crisis

The FDA has approved l-glutamine and crizanlizumab for SCD to reduce the frequency of VOC, they are not for treating VOC in the acute setting to prevent the development of sVOC. To be beneficial in treating VOC, an agent has to be able to mediate its effects promptly after administration to interrupt the vicious cycle of VOC to prevent sVOC. Clinical trials investigating specific agents for treating VOC have focused on targeting the downstream events and have uniformly been disappointing. This is not surprising taking into consideration that VOC is a complex pathologic process and leads to many downstream events. Currently, the mainstay of management in SCD patients with acute VOC is intravenous hydration and opioid analgesia. This conservative approach aims to treat the patients symptomatically and wait for the VOC process to resolve spontaneously. In this section, we will discuss some of the therapeutic agents that may be investigated for use during acute VOC to either protect erythrocytes that are not already involved in the hemoglobin polymerization process from undergoing sickling, or downregulate the downstream effects of VOC.

### Anti-sickling agents

Even during an acute VOC episode, only a population of HbS erythrocytes are affected by the sickling process. It, therefore, follows that protecting or “fortifying” the un-involved HbS erythrocytes from sickling and subsequent sVOC is a rational and probably most effective approach since this would provide protection without having to address the multi-pathway secondary events. HbS erythrocytes can be prevented from sickling by increasing the oxygen affinity of SCD erythrocytes. Although increasing the fetal hemoglobin that has a high affinity for oxygen using hydroxyurea [48], DNA hypomethylating agents [49], or phosphodiesterase 9 inhibitors [50] prevents sickling, such approaches are not relevant in the acute setting to prevent sVOC. However, other strategies might be explored. Increasing the oxygen affinity of HbS using the allosteric modifier, voxelotor [51], is a very attractive option since voxelotor binds directly to HbS to modify its oxygen affinity rapidly. Pharmacokinetic studies support the feasibility of this approach since the maximum concentration in plasma is reached within two hours and whole blood in six hours [52] following an oral dose of voxelotor. It should, however, be pointed out that voxelotor did not manage to reduce the frequency of VOC in a randomized study [51].

*N*-acetylcysteine (NAC) reduces erythrocyte oxidative stress [53] and prevents sickling of HbS erythrocytes. Although a Phase 3 placebo-controlled randomized study failed to support its use to reduce the frequency of VOC [54], its role in treating VOC and preventing sVOC has not been examined. Pharmacokinetic studies of NAC would support its potential role in the acute setting. Its maximum concentration in plasma can be reached between half and three hours after one single oral dose [55]. NAC is currently not licensed for use in SCD.

Other approaches that may improve the oxygen affinity of HbS erythrocytes in the acute setting that are currently being investigated include the use of pyruvate kinase activators [56, 57]. These agents stimulate the glycolytic pathway to deplete the intra-erythrocyte 2,3-diphosphoglycerate.

### Anti-inflammatory agents

Since intense inflammatory processes are associated with VOC, the use of anti-inflammatory agents may be appropriate. Although it is the gold standard for treating SCD, less than 30% of SCD patients are on long-term hydroxyurea [58]. Therefore, a high proportion of SCD patients admitted to the hospital are not already on the medication. Its use during active VOC will not result in induction of HbF. However, hydroxyurea mediates anti-inflammatory processes associated with SCD [59, 60], probably via downregulation of STAT3 and may prevent the development of sVOC. Furthermore, the rapid onset of myelosuppression, usually within 36–48 h, when used in high doses may also provide benefits to reduce the pool of activated and aged neutrophils that participate in VOC and sVOC. The use of high dose urea during acute VOC is being investigated (Clinicaltrial.gov: NCT03062501).

Anti-inflammatory agents may prevent the development of sVOC. Although NSAID such as ketorolac has been incorporated in the pain management of SCD [61], there has not been any studies demonstrating their use in preventing sVOC and shortening the duration of the crisis. They should also be used with caution in view of the high frequency of renal impairment in this group of patients. On the other hand, high dose intravenous methylprednisolone to shortened the duration of hospital stay compared to those on the placebo arm, although the patients were more likely to be readmitted to the hospital after discharge due to rebound toxicity [62]. These results support the role of corticosteroids in the treatment of VOC but how they should be used to reduce the likelihood of hospital readmissions remains to be established.

### Agents targeting adhesion

Adherence of sickle erythrocytes to the endothelium occurs either directly, or more often, via activated leukocytes adherent to the endothelium. Targeting cellular adhesion molecules involved in their interaction, once VOC occurs, will reduce the likelihood of development of sVOC. Crizanlizumab, a monoclonal antibody against P-selectin [63] and has been approved by the FDA for use to reduce the frequency of VOC, may be a suitable agent to treat VOC to prevent sVOC. On the other hand, rivipansel, a pan-selectin antibody, has been shown in a Phase 2 study to shorten the duration of VOC compared to placebo [64], although the results (yet to be published) from its Phase 3 study failed to meet both its primary and secondary endpoints. Investigations on the role of antibodies directed at iNKT cells activated by ischemia–reperfusion injury to treat VOC, unfortunately, failed to show benefits [65].

Based on the laboratory results showing the ability of IVIg to reverse acute VOC in SCD mice [66, 67], a Phase 1/2 study was performed with a fixed dose of IVIg at 800 mg/kg. Unfortunately, the laboratory successes could not be reproduced in the clinic [68]. It may be worthwhile exploring a higher dose of IVIg, although it will have to be administered carefully due to the high sodium load on the renal functions.

### Modulators of intestinal pathology

As discussed above, SCD is associated with intestinal pathology that contributes to the chronic inflammatory processes and the development of VOC. Targeting the intestinal pathology during VOC may, therefore, reduce the likelihood for the development of sVOC. Intestinal barrier in SCD may be improved by l-glutamine that enhances enterocyte health and the development of tight junctions [69]. l-glutamine is approved by the FDA for treating SCD based on a randomized study showing reduction in the frequency of VOC in those treated with l-glutamine, compared to placebo [25], although the mechanisms responsible for the clinical benefits remain to be determined. l-glutamine use during VOC episodes may restore intestinal barrier and reduce the bacteria/bacterial product translocation.

Based on a small study showing that the antibiotic rifaximin reduced the frequency of VOC, rifaximin [27] may also be tried during VOC episodes to reduce the intestinal microbial load and prevent sVOC. Rifaximin use in the preventive setting reduce the number of CANs and serum LPS [28]. Antibiotic use may also downregulate the VOC due to microbiota-mediated expansion of CANs [47] induced by the stress due with pain during VOC.

### Modulators of VOC pathway induced by stress

VOC is associated with intense sympathetic responses due to pain. As discussed above, epinephrine upregulates the expression of adhesion molecules on erythrocytes [45] to promote VOC. In addition, the augmented glucocorticoid responses increase gut permeability and promotes microbiota-dependent IL-17A secretion [47], leading to the precipitation of VOC. Downregulation of the sympathetic responses may interrupt the vicious cycle of VOC. This may be accomplished with the use of β-blocker such as propranolol [70], or more appropriately, with aggressive analgesia treatment. Early achievement of maximum analgesia improved hospitalization outcomes [71].

### Nitric oxide facilitators

One of the hallmarks of SCD, especially during VOC episodes, is the depletion of nitric oxide (NO). NO mediates vaso-dilatation. NO depletion causes vaso-constriction and increases the risks for VOC. Enhancing NO availability during VOC is, therefore, rational and may prevent sVOC. NO availability can be derived from inhaled NO during VOC or use of agents such as l-citrulline [72] and l-arginine [73], both are substrates in the NO cycle. Clinical studies using inhaled NO monotherapy to treat VOC, however, did not find any obvious clinical benefits [74, 75].

### Modulators of blood viscosity

The rationale for the administration of intravenous fluid to SCD patients during VOC episode is to treat any underlying dehydration that could have precipitated the VOC, reduce the blood viscosity to prevent the development of sVOC, and perhaps, dilute the inflammatory cytokines associated with VOC. Unless contraindicated or with a history of fluid overload that occurs not infrequently in SCD patients [76], these patients should receive aggressive intravenous fluid with hypotonic and low sodium fluid to reduce the sodium intake since SCD patients have reduced ability to excrete sodium load [77].

As a result of NETosis, the DNA strands increases blood viscosity and stimulate the coagulation cascade [41] that would feed into the vicious cycle of VOC to induce sVOC. Administration of DNAse to SCD mice has produced beneficial effects of VOC [20]. Such an approach might be investigated in future in human using recombinant human DNAse.

### Antiplatelets and anticoagulation therapy

VOC activates platelets and the coagulation cascade. Use of antiplatelet and anticoagulating agents during acute VOC episodes is, therefore, rational. Unfortunately, their use individually as monotherapy for treating VOC has so far not produced significant benefits [78–81].

## Conclusions

**Fig. 3:**
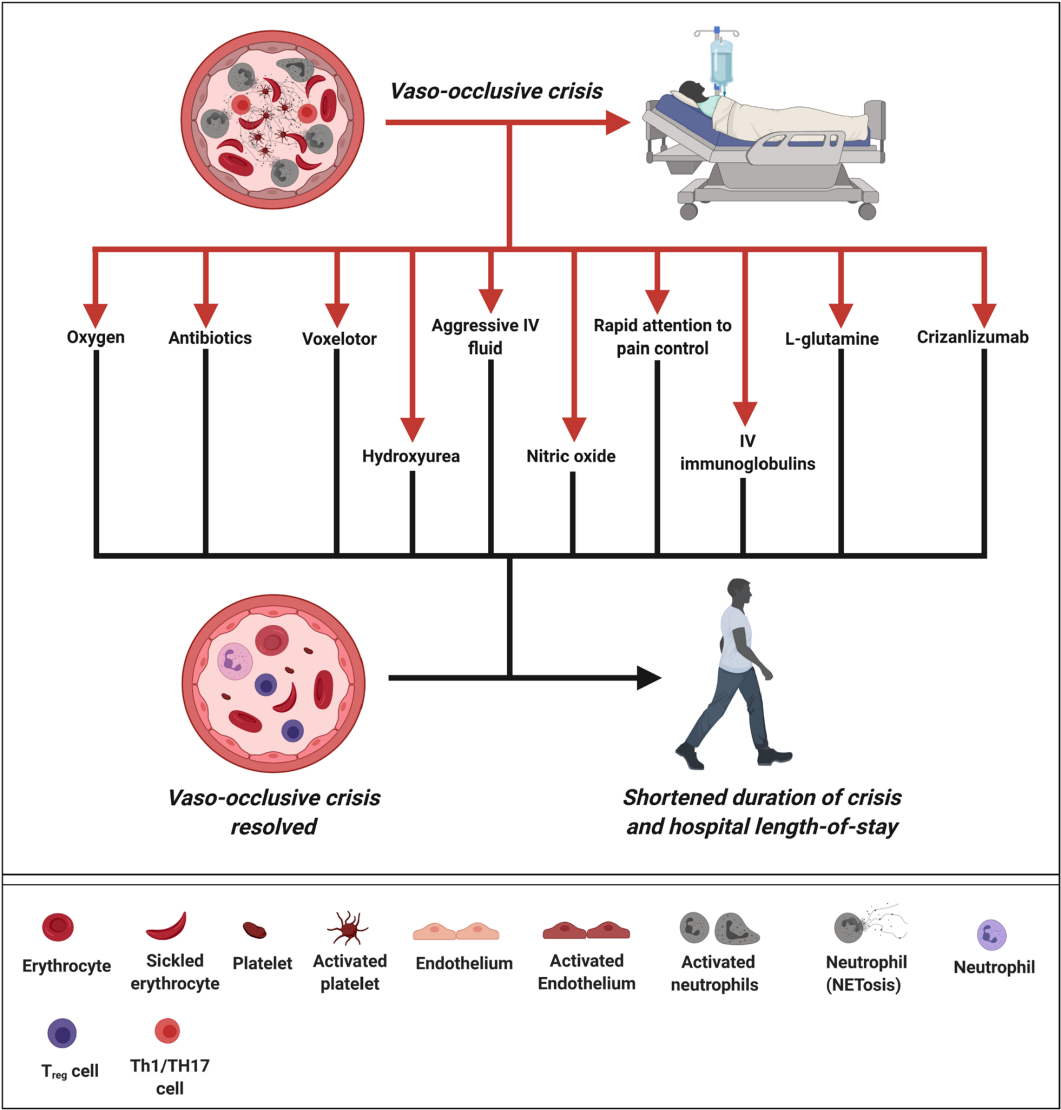
A proposed multipronged approach to treating VOC. Instead of just using intravenous fluid, oxygen, and pain management and wait for the VOC process to resolve spontaneously, a more proactive approach is proposed using a combination of the listed agents to target as many of the downstream processes as possible. Acute use of antisickling agents such as voxelotor may protect the un-involved erythrocytes from sickling induced by the many downstream events of VOC. On the other hand, approaches that target the downstream events will more likely be successful if used in combination to overcome as many of the downstream pathways as possible

While advances have been made to reduce the frequency of VOC in SCD, there have been very little change in the management of patients during episodes of VOC. The current passive approach relies solely on supportive measures and wait for the spontaneous resolution of the VOC episodes. As a result, the duration of VOC is prolonged in patients who are caught in the vicious cycle of the pathology with the development of sVOC. A more aggressive approach that would treat the VOC instead is needed. The results from various studies on treating VOC using monotherapy have so far been universally disappointing. This is not surprising in view of the complex nature of VOC. Unless targeting the erythrocytes to protect the un-involved HbS erythrocytes from secondary sickling, successful treatment of VOC targeting of the downstream pathways requires multipronged approaches using combination therapy, just like treatment of malignant diseases using combination chemotherapy. Based on these arguments, it is possible that treatment of acute VOC to prevent sVOC may only be efficacious using monotherapy with agents that increase the oxygen affinity acutely to prevent further sickling, or combination therapy that targets multiple downstream processes. Figure 3 represents a multimodality approach based on the agents currently available in the market that could be investigated in various combinations that cover as many of the different downstream events as possible to prevent the development of sVOC. The combination of approaches targeting more than one downstream pathway may shorten the duration of VOC and reduce the hospital LOS so that the patient can be discharged and managed on oral analgesia.

## Data Availability

N/A.

## Abbreviations

BACH: BTB and CNC homologue
CANs: Circulating aged neutrophils
CD: Cluster of differentiation
DNA: Deoxyribonucleic acid
ED: Emergency department
FITC: Fluorescein isothiocyanate
Hb: Hemoglobin
HbF: Fetal hemoglobin
HbS: Sickle hemoglobin
ICAM: Intercellular adhesion molecules
iFABP: Intestinal fatty acid binding proteins
IL: Interleukin
iNKT: Invariant natural killer T
IVIg: Intravenous immunoglobulins
LOS: Length-of-stay
LPS: Lipopolysaccharides
Myd: Myeloid differentiation
NAC: *N*-Acetylcysteine
NETosis: Neutrophil extracellular traps
NSAID: Non-steroidal anti-inflammatory drug
NO: Nitric oxide
ROS: Radical oxygen species
SCD: Sickle cell disease
STAT: Signal transducer and activator of transcription
sVOC: Secondary vaso-occlusive crisis
TLR: Toll-like receptor
TNF: Tumor necrosis factor
VOC: Vaso-occlusive crisis
